# Effects of Deoxynivalenol and Mycotoxin Adsorbent Agents on Mitogen-Activated Protein Kinase Signaling Pathways and Inflammation-Associated Gene Expression in Porcine Intestinal Epithelial Cells

**DOI:** 10.3390/toxins13050301

**Published:** 2021-04-23

**Authors:** Yu-Hsiang Yu, Yi-Han Lai, Felix Shih-Hsiang Hsiao, Yeong-Hsiang Cheng

**Affiliations:** 1 Department of Biotechnology and Animal Science, National Ilan University, Yilan 26047, Taiwan; yuyh@niu.edu.tw (Y.-H.Y.); laiyihan04@outlook.com (Y.-H.L.); 2 Department of Animal Science and Biotechnology, Tunghai University, Taichung 407224, Taiwan; hsh@thu.edu.tw

**Keywords:** deoxynivalenol, mitogen-activated protein kinase, mycotoxin adsorbent agent, inflammation, porcine intestinal epithelial cell

## Abstract

Deoxynivalenol (DON) is the most prevalent mycotoxin in swine feedstuffs. The intestinal epithelial cells represent the first target for the DON. Here, we studied the effects of DON and mycotoxin adsorbent agents on mitogen-activated protein kinase (MAPK) signaling pathways and inflammation-associated gene expression in porcine intestinal epithelial cells (IPEC-J2). Results showed that phosphorylation of MAPK signaling pathways (p38, ERK, and JNK) was increased after treatment of DON or lipopolysaccharide (LPS) in IPEC-J2 cells. The phosphorylation of p38, ERK, and JNK was not further enhanced after co-treatment with DON and LPS. The *inos* and *cox-2* mRNA expression were significantly induced at 6 h after treatment of DON. DON treatment significantly increased the *claudin 3* and *occludin* mRNA expression at 12 h. DON in combination with LPS treatment did not further increase the inflammation and tight junction-associated gene expression. The DON-induced phosphorylation of MAPK signaling pathways was impaired by mycotoxin adsorbent agent (nanoscale silicate platelets and the mixture of montmorillonites and yeast cell walls) treatment, thereby decreasing inflammation and tight junction-associated gene expression. Taken together, these findings demonstrate that DON triggers the inflammation in IPEC-J2 cells by phosphorylation of MAPK signaling pathways and LPS does not further augment the DON-induced inflammatory responses. Mycotoxin adsorbent agents can attenuate DON-induced inflammatory responses in IPEC-J2 cells through modulation of the phosphorylation of p38, ERK, and JNK.

## 1. Introduction

The provision of feed is an important source of nutrients for animal production. However, fungal contamination of animal feed is a global epidemic that causes a significant economic impact on animal health [1]. Mycotoxin, the secondary metabolites secreted by molds, exhibits acute and chronic effects on livestock depending on species and susceptibility [2]. Among mycotoxins, deoxynivalenol (DON) is the most common mycotoxin in the feed and cereal-based feed ingredients [3,4]. Pigs are considered highly susceptible to DON contamination versus other farm animals [5]. Exposure of the pigs to high levels of DON leads to a reduced body weight gain and feed intake [6]. A low level of DON causes immune system dysfunction and alters the intestinal barrier in pigs [7,8].

The gastrointestinal tract, a critical interface between the body and ingested nutrients, plays an important role in the prevention of pathogen invasion. Intestinal epithelial cells are the main physical barrier between cells connected by protein complexes, such as gap junctions, tight junctions, and adherens junctions, which maintain intestinal barrier integrity and tightness [9]. The previous study indicates that DON is able to attenuate the expression of tight junction protein by phosphorylation of the extracellular signal-regulated kinase (ERK) signaling pathway, resulting in impairing intestinal integrity [10,11]. Chronic or acute exposure to DON activates inflammation in intestinal epithelial cells, which leads to disrupt the intestinal barrier function [12]. DON also potentiates intestinal inflammation in response to pathogen challenge in pigs [13]. Chronic and low doses of DON ingestion induce inflammatory genes and inhibit tight junction gene expression in the intestine [14,15]. Bacterial lipopolysaccharide (LPS) is widely present in the porcine gastrointestinal tract and is able to amplify DON toxicity in mice [16]. LPS alone also triggers inflammatory signaling and impairs intestinal integrity in porcine intestinal epithelial cells [17,18]. Thus, the gastrointestinal tract represents the first barrier to DON and LPS.

Several chemical and physical strategies have been investigated for the detoxification of mycotoxin-contaminated feeds [19]. Among these methods, the mycotoxin adsorbent is one of the efficient and widely used strategies to reduce the risk for mycotoxicoses in livestock [20]. It has been demonstrated that montmorillonite has the potential for the prevention of DON contamination in feeds [21]. Nanoscale silicate platelet (NSP), an exfoliated product from montmorillonites, is able to adsorb fumonisin B1 and exhibits no adverse effect on the murine embryo development in vitro [22]. Besides, yeast cell walls can bind several mycotoxins in vitro, such as zearalenone, aflatoxin B1, and ochratoxin A [23,24].

Mitogen-activated protein kinase (MAPK) signaling pathways, including p38, ERK, and c-Jun N-terminal kinase (JNK) mediate inflammatory gene and cytokine expression [25]. It has been demonstrated that DON activates ERK phosphorylation and decreases tight junction protein expression in IPEC-1 cells [10]. MAPK signaling pathways are induced in response to DON stimulation in IPEC-J2 cells, which was accompanied by an increase in inflammatory gene expression [26]. DON-induced inflammation can be abolished by p38 and ERK inhibitor in IPEC-J2 cells [26], indicating that both p38 and ERK play a critical role in the regulation of DON-induced inflammatory gene and cytokine expression in porcine intestinal epithelial cells.

It is still unclear whether LPS can potentiate DON-induced inflammation in the porcine intestinal epithelial cells by distinct regulatory pathways. Further, whether nanoscale silicate platelet and montmorillonites in combination with yeast cell walls attenuate the adverse effect of DON on porcine intestinal epithelial cells is rarely investigated. To our knowledge, no one has investigated the effects of DON in combination with LPS or mycotoxin adsorbent agents on MAPK signaling pathways and inflammation-associated gene expression on porcine intestinal epithelial cell line IPEC-J2. We hypothesized that the DON-induced inflammatory response on porcine intestinal epithelial cells can be reversed by mycotoxin adsorbent agents. Therefore, the current study aimed to explore the effect of DON and mycotoxin adsorbent agents on MAPK signaling pathways and inflammation-associated gene expression in the intestinal epithelial cell line IPEC-J2.

## 2. Results

### 2.1. Effect of Deoxynivalenol and Lipopolysaccharide on Phosphorylation of MAPK Signaling Pathways and Inflammation and Tight Junction-Associated Gene Expression

**Figure 1:**
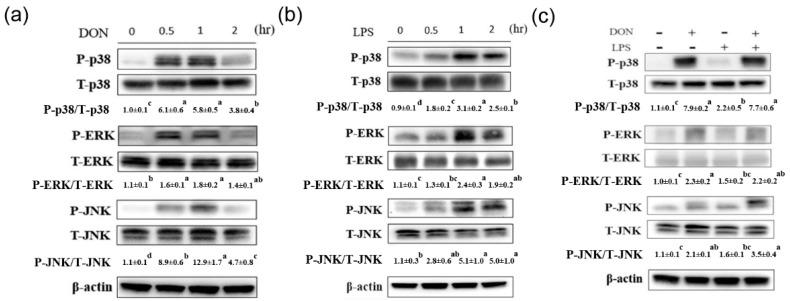
Deoxynivalenol and lipopolysaccharide on inflammatory signaling pathways in IPEC-J2 cells. Cells were treated with (**a**) deoxynivalenol (DON, 1 µg/mL), (**b**) lipopolysaccharide (LPS, 100 ng/mL), or (**c**) both at the indicated times (0.5, 1, and 2 h). Three independent experiments were performed (*n* = 3), and one representative result is presented. The numbers indicate the means ± standard deviation. Different superscripts mean statistically different.

**Figure 2:**
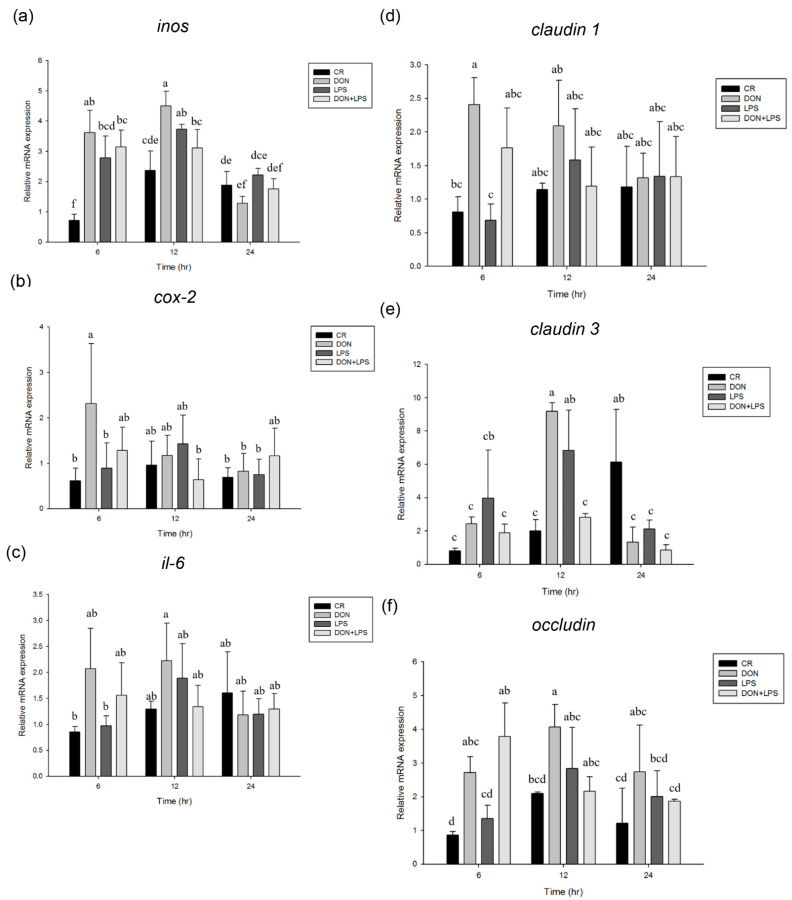
Effect of deoxynivalenol and lipopolysaccharide on inflammation and tight junction-associated gene expression in IPEC-J2 cells. Effects of control (CR), deoxynivalenol (DON), lipopolysaccharide (LPS), or both (DON+LPS) on (**a**) *inos*, (**b**) *cox-2*, and (**c**) *il-6* gene expression at the indicated times (6, 12, and 24 h). Effects of control (CR), DON, LPS, or both (DON+LPS) on (**d**) *claudin 1*, (**e**) *claudin 3*, and (**f**) *occludin* gene expression at the indicated times (6, 12, and 24 h). Three independent experiments were performed (*n* = 3). Each bar represents mean ± standard deviation. Different superscripts mean statistically different.

The effect of DON on phosphorylation of MAPK signaling pathways in IPEC-J2 cells is shown in Figure 1a. Results showed that the phosphorylation of p38 reached their maximum levels at 0.5 h and then declined at 2 h after treatment of DON (*p* < 0.05). Similarly, the phosphorylation of ERK reached a peak at 0.5 h (*p* < 0.05) and declined to the basal level at 2 h after treatment of DON. The increase of JNK phosphorylation was observed after 0.5 h treatment with DON (*p* < 0.05) and peak JNK phosphorylation level was reached at 1 h after DON treatment (*p* < 0.05). The effect of LPS on phosphorylation of signaling transduction pathways in IPEC-J2 cells is shown in Figure 1b. The phosphorylation of p38 was induced at 0.5 h (*p* < 0.05) and reached a peak at 1 h after treatment of LPS (*p* < 0.05). The phosphorylation of ERK and JNK reached their maximum levels at 1 h and then declined at 2 h in response to LPS stimulation (*p* < 0.05). The effect of co-treatment with DON and LPS on phosphorylation of signaling transduction pathways in IPEC-J2 cells is shown in Figure 1c. Compared with LPS, DON strongly enhanced the phosphorylation of p38, ERK, and JNK (*p* < 0.05). The phosphorylation of p38, ERK, and JNK was not further enhanced after co-treatment with DON and LPS. In inflammation-associated gene expression, *inos* mRNA expression was induced at 6 h after treatment of DON, LPS, or both (*p* < 0.05, Figure 2a). DON or LPS treatment for 12 h could increase the *inos* mRNA expression in IPEC-J2 cells compared with untreated cells (*p* < 0.05). The expression of *cox-2* mRNA was induced at 6 h after treatment of DON (*p* < 0.05, Figure 2b), whereas LPS alone or LPS in combination with DON treatment did not affect the *cox-2* mRNA expression. Compared with 6 h, *il-6* mRNA expression was induced at 12 h after treatment of DON (*p* < 0.05, Figure 2c). In tight junction-associated gene expression, DON treatment for 6 h could increase the *claudin 1* mRNA expression in IPEC-J2 cells compared with untreated cells (*p* < 0.05, Figure 2d). The expression of *claudin 3* mRNA was induced at 12 h after treatment of DON or LPS (*p* < 0.05, Figure 2e), whereas this effect was abolished when cells were simultaneously treated with DON and LPS. DON or DON in combination with LPS treatment for 6 h could induce the *occludin* mRNA expression (*p* < 0.05, Figure 2f). The expression of *occludin* mRNA was induced at 12 h after treatment of DON (*p* < 0.05, Figure 2f), whereas this effect was abolished when cells were simultaneously treated with DON and LPS.

### 2.2. Effect of Deoxynivalenol in Combination with Mycotoxin Adsorbent Agents on Phosphorylation of MAPK Signaling Pathways and Inflammation and Tight Junction-Associated mRNA Expression

**Figure 3:**
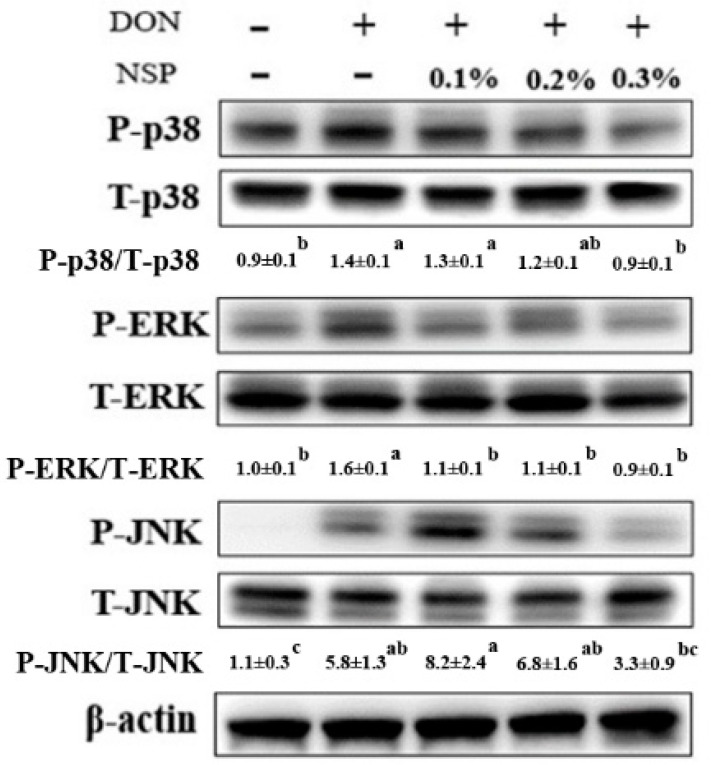
Effect of the mixture of deoxynivalenol and nano silicate platelets on inflammatory signaling pathways in IPEC-J2 cells. Cells were treated with the mixture of deoxynivalenol (DON, 1 µg/mL) and nano silicate platelets (NSP, 0.1–0.3%) for 1 h. Three independent experiments were performed (*n* = 3), and one representative result is presented. The numbers indicate the means ± standard deviation. Different superscripts mean statistically different.

**Figure 4:**
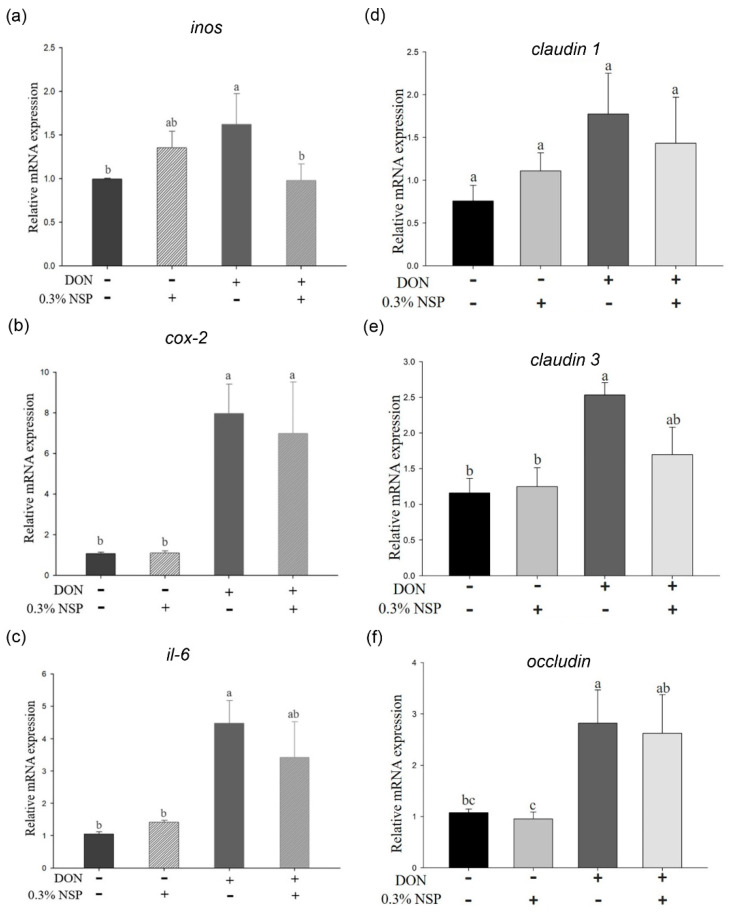
Effect of the mixture of deoxynivalenol and nano silicate platelet on inflammation and tight junction-associated gene expression in IPEC-J2 cells. Cells were treated with the mixture of deoxynivalenol (DON, 1 µg/mL) and nano silicate platelets (NSP, 0.3%) for 6 h. The (**a**) *inos*, (**b**) *cox-2*, and (**c**) *il-6* mRNA expression were analyzed by quantitative reverse transcription PCR. Cells were treated with the mixture of DON and NSP for 12 h. The (**d**) *claudin 1*, (**e**) *claudin 3*, and (**f**) *occludin* mRNA expression were analyzed by quantitative reverse transcription PCR. Three independent experiments were performed (*n* = 3). Each bar represents mean ± standard deviation. Different superscripts mean statistically different.

The effect of DON in combination with nanoscale silicate platelet on phosphorylation of signaling transduction pathways in IPEC-J2 cells is shown in Figure 3. The phosphorylation of p38 was activated in cells after treatment of DON (*p* < 0.05), whereas 0.3% NSP treatment could alleviate the phosphorylation of p38 in DON-treated cells. Similarly, the ERK and JNK phosphorylation was induced by DON in IPEC-J2 cells (*p* < 0.05), whereas 0.3% NSP treatment could reduce the ERK and JNK phosphorylation in DON-treated cells. In inflammation-associated gene expression, DON induced the *inos* mRNA expression (*p* < 0.05, Figure 4a), whereas 0.3% NSP treatment alleviated the *inos* mRNA expression in DON-treated cells (*p* < 0.05, Figure 4a). However, DON in combination with 0.3% NSP treatment did not reduce the mRNA expression of *cox-2* and *il-6* compared with DON treatment alone (Figure 4b,c). In tight junction-associated gene expression, there was no significant difference in *claudin 1* mRNA expression among groups (Figure 4d). The *claudin 3* and *occludin* mRNA expression was significantly induced by DON treatment (*p* < 0.05, Figure 4e,f). DON in combination with 0.3% NSP treatment did not reduce the mRNA expression of *claudin 3* and *occludin* in IPEC-J2 cells compared with DON treatment alone (Figure 4e,f). 

**Figure 5:**
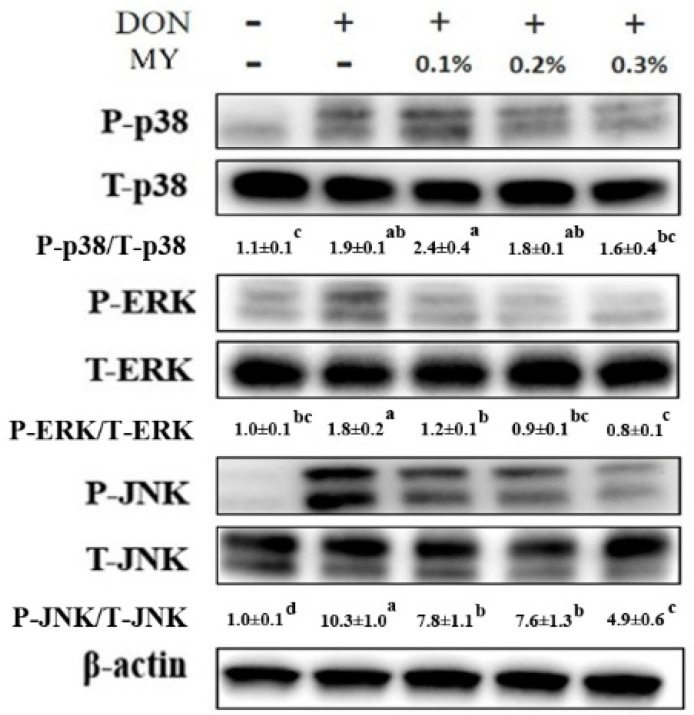
Effect of the mixture of deoxynivalenol and montmorillonites in combination with yeast cell walls on inflammatory signaling pathways in IPEC-J2 cells. Cells were treated with the mixture of deoxynivalenol (DON, 1 µg/mL) and montmorillonites in combination with yeast cell walls (MY, 0.1–0.3%) for 1 h. Three independent experiments were performed (n = 3), and one representative result is presented. The numbers indicate the means ± standard deviation. Different superscripts mean statistically different.

**Figure 6:**
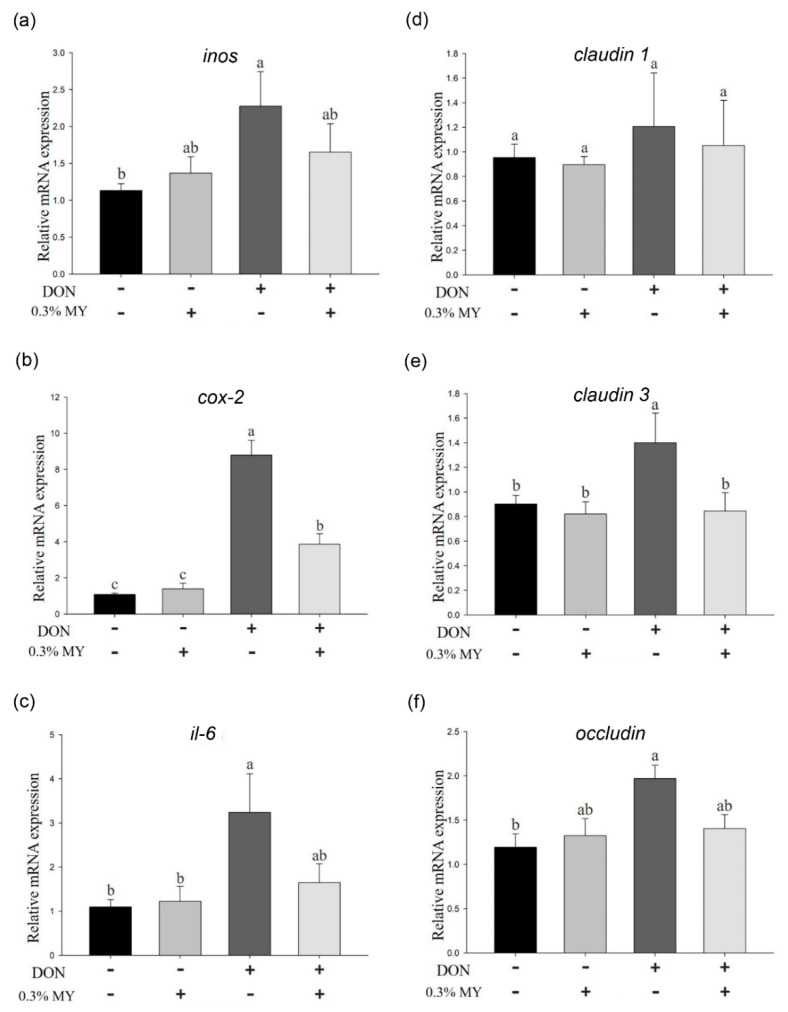
Effect of the mixture of deoxynivalenol and montmorillonites in combination with yeast cell walls on inflammation and tight junction-associated gene expression in IPEC-J2 cells. Cells were treated with the mixture of deoxynivalenol (DON, 1 µg/mL) and montmorillonites in combination with yeast cell walls (MY, 0.3%) for 6 h. The (**a**) *inos*, (**b**) *cox-2*, and (**c**) *il-6* gene expression were analyzed by quantitative reverse transcription PCR. Cells were treated with the mixture of DON and MY for 12 h. The (**d**) *claudin 1*, (**e**) *claudin 3*, and (**f**) *occludin* gene expression were analyzed by quantitative reverse transcription PCR. Three independent experiments were performed (*n* = 3). Each bar represents mean ± standard deviation. Different superscripts mean statistically different.

The phosphorylation of p38, ERK, and JNK was induced in IPEC-J2 cells after treatment of DON (*p* < 0.05), whereas 0.3% MY treatment could alleviate the phosphorylation of p38, ERK, and JNK in DON-treated cells (Figure 5). The *inos*, *cox-2*, and *il-6* mRNA expression was significantly induced by DON treatment (*p* < 0.05, Figure 6a–c). 0.3% MY treatment did not affect the mRNA expression of *inos* and *il-6* in DON-treated cells (Figure 6a,c). In contrast, 0.3% MY treatment alleviated the mRNA expression of *cox-2* in DON-treated cells (*p* < 0.05, Figure 6b). In tight junction-associated gene expression, no significant difference was found in *claudin 1* mRNA expression among groups (Figure 6d). The *claudin 3* and *occludin* mRNA expression was significantly induced by DON treatment (*p* < 0.05, Figure 6e), whereas 0.3% MY treatment could inhibit the *claudin 3* mRNA expression in DON-treated cells (*p* < 0.05, Figure 6e). DON treatment in IPEC-J2 cells significantly increased *occludin* mRNA expression (*p* < 0.05, Figure 6f). However, DON in combination with 0.3% MY treatment in IPEC-J2 cells did not reduce the mRNA expression of *occludin* compared with DON treatment alone (Figure 6f).

## 3. Discussion

In the present study, we demonstrated that DON or LPS treatment induced phosphorylation of p38, ERK, and JNK in IPEC-J2 cells. The phosphorylation of JNK was further enhanced after co-treatment with DON and LPS. DON treatment increased the mRNA expression of *inos*, *cox-2*, *claudin 3*, and *occludin* in IPEC-J2 cells. DON in combination with LPS treatment did not further increase the inflammation and tight junction-associated gene expression. DON-induced phosphorylation of MAPK signaling pathways was attenuated by mycotoxin adsorbent agent treatment, thereby decreasing inflammation and tight junction-associated gene expression.

Previous studies have reported that DON impairs intestinal morphology and induces an inflammatory response in the gut of pigs [8,14]. Chronic ingestion of DON (3 mg/kg of feed) for 5 weeks increases inflammatory gene expression in the ileum or the jejunum of piglets, including *tnf-α*, *il-1β*, *il-6*, *il-10*, and *ifn-γ* [14]. The mRNA expression of *cox-2*, *il-1β*, and *il-10* is elevated in the gut of growing pigs after low-dose and short-term DON exposure (0.9 mg/kg of feed for 10 days) [8]. It has been demonstrated that MAPK signaling pathways, including p38, ERK, and JNK play a critical role in the regulation of inflammatory gene and cytokine expression [25]. At the cellular level, it has been suggested that DON activates ERK phosphorylation and decreases tight junction protein expression in IPEC-1 cells [10]. Furthermore, p38, ERK, and JNK phosphorylation are induced in response to DON stimulation in IPEC-J2 cells, which was accompanied by an increase in inflammatory gene expression [26]. Nuclear factor κB (NF-κB), a downstream transcription factor of MAPK signaling pathways, is a critical transcription factor controlling inflammatory and immune responses. The protein expression and phosphorylation of NF-κB is induced in IPEC-J2 cells after DON treatment [27]. Here, the phosphorylation of MAPK signaling pathways and inflammatory gene expression was also increased in IPEC-J2 cells after treatment of DON, which is in agreement with previous studies [25,26,27,28]. In addition to DON, it has been shown that low concentrations of LPS can be detected in healthy pigs without triggering systemic inflammation [29]. LPS also activates the phosphorylation of MAPK signaling pathways, thereby inducing an inflammatory response in IPEC-J2 cells [30]. Similar to a previous study [30], the phosphorylation of MAPK signaling pathways and inflammatory gene expression was also increased in IPEC-J2 cells after treatment of LPS in the present study. Although it has been reported that amplified inflammatory cytokine expression is observed in mice exposed to DON and LPS [16]. Here, DON did not interact with LPS on phosphorylation of MAPK signaling pathways and inflammatory gene expression in IPEC-J2 cells. This observation is in agreement with the results of Klunker et al. [31], who observed no synergistic effect of DON and LPS on intestinal cell proliferation and morphology in pigs. It could be possible that DON and LPS promote inflammatory response through a similar mechanism since DON and LPS all activate MAPK signaling pathways (p38, ERK, and JNK). Collectively, DON treatment is able to induce inflammatory responses in the porcine intestinal epithelial cells through activation of MAPK signaling pathways. Whether MAPK signaling pathways also regulate the intestinal inflammatory response in pigs exposed to DON remains to be confirmed.

Tight junction proteins, such as occludin and claudin, are involved in intestinal epithelium formation and development, the maintenance of integrity, and regulation of permeability. Previous studies have indicated that occludin protein expression is reduced in the gut of piglets in response to chronic and long-term DON exposure [14]. In another study, occludin protein expression in different parts of the intestine (duodenum, jejunum, and colon) is induced in growing pigs exposed to low-dose and short-term DON [8]. The mRNA levels of *claudin 4* and *occludin* are decreased in the jejunum in DON-treated growing pigs, whereas mRNA levels of *claudin 1*, *claudin 3*, *claudin 4*, and *occludin* are elevated in the ileum, cecum, and colon [8]. At the cellular level, the claudin 4 protein expression is attenuated in DON-treated IPEC-1 cells [10]. DON also decreases tight junction-associated protein expression in the IPEC-J2 cells [32]. In contrast, tight junction-associated gene expression (*claudin 1*, *claudin 3*, and *occludin*) was increased at 6 and 12 h after treatment of DON in the IPEC-J2 cells in the present study. The *claudin 1* and *claudin 3* mRNA levels were then reduced at 24 h after treatment of DON compared with 6 or 12 h, indicating that *claudin 1* and *claudin 3* gene was dynamically regulated in response to DON treatment. A dynamic gene expression of *claudin 1* and *occludin* is also observed in the IPEC-J2 cells depends on DON concentration and exposure time [28]. It has been reported that DON is mainly absorbed in the upper small intestine, whereas the large intestine is exposed to DON via the bloodstream [33]. DON can trigger different gene responses between apical and basolateral intestinal epithelial cells [11]. Furthermore, MAPK signaling pathways discriminatively modulate the tight junction-associated protein expression in the murine proximal epididymis [34]. Taken together, these findings indicated that tight junction-associated genes and proteins are differentially regulated by DON in the different concentrations, exposure time, parts of the intestine, and cell lines.

Several physical, chemical, and biological strategies have been developed for decontaminating mycotoxins in contaminated feed [35]. Among these materials, montmorillonite, a clay mineral composed of aluminosilicate, is a highly effective adsorbent for removing toxic compounds from air, soil, and water. Due to the physical–chemical property, montmorillonite, as mycotoxin adsorbent agents, is one of the efficient strategies for the reduction of adverse effects induced by mycotoxin-contaminated feeds in pigs [21]. Many studies have confirmed the detoxification properties of montmorillonite against mycotoxins [36,37]. It has been demonstrated that montmorillonite causes a significant improvement in growth performance in piglets fed aflatoxin-contaminated diets [38,39]. Low levels of zearalenone in the diet impair nutrient digestibility and growth in piglets, whereas montmorillonite can reverse the adverse effects caused by zearalenone [40]. NSP, an exfoliated product from montmorillonites, is able to adsorb fumonisin B1 and has no adverse effect on murine embryo development and epithelial cell proliferation in vitro [22,41]. NSP ameliorates fumonisin B1 toxicosis and growth performance in broilers [42]. These animal model results suggest that NSP is safe and has beneficial effects on the detoxification of mycotoxins, although the effect of NSP on DON detoxification is still unclear. Yeast cell wall, rich in mannan-oligosaccharide and β-glucan, is also used as a mycotoxin adsorbing agent in the animal feed [23,24]. It has been demonstrated that the removing ability for DON of yeast cell wall product was greater than montmorillonite [43]. Dietary supplementation of the yeast cell wall can partially reduce the adverse effects of DON on the immune system of pigs [44]. The chronic dietary challenge of DON in combination with aflatoxin B1 decreases the growth and nutrient digestibility in weaning piglets, whereas supplementation with the yeast cell wall can partially overcome the harmful effects of mycotoxin challenge [45]. These studies imply that yeast cell wall has beneficial effects on detoxification of DON in pigs. At the cellular level, it has been demonstrated that montmorillonite or yeast cell wall suppresses DON-induced inflammatory response in IPEC-1 cells by downregulation of MAPK phosphorylation [46]. To our best knowledge, we are the first to investigate the effect of NSP and MY on DON-induced phosphorylation of MAPK signaling pathways and inflammation and tight-junction associated gene expression in the well-differentiated and morphologically highly representative IPEC-J2 cells. We are therefore the first to confirm that DON-induced phosphorylation of p38, ERK, and JNK was impaired after pre-incubation with mycotoxin adsorbent agents (NSP and MY), thereby decreasing inflammation and tight junction-associated gene expression, including *inos*, *cox-2*, and *claudin 3*. This observation is in agreement with the results of Park et al. [46], who observed that montmorillonite or yeast cell wall can attenuate DON-induced *il-8* gene expression in IPEC-1 cells by downregulation of p38 and ERK phosphorylation. These findings further support that DON regulates the inflammation and tight junction-associated gene expression by activation of MAPK signaling pathways. In future research, the DON-mediated MAPK signaling pathway activation in the porcine gastrointestinal tract remains to be confirmed in vivo. Whether dietary supplementation of NSP or MY in pigs can reduce the adverse actions caused by DON on growth performance, inflammatory response, and gut morphology still needs to be verified.

## 4. Conclusions

We provide evidence that DON triggers the inflammatory response in IPEC-J2 cells and LPS does not further augment the DON-induced inflammatory responses. Mycotoxin adsorbent agents have a suppressive effect on DON-induced inflammation in IPEC-J2 cells by modulation of the MAPK signaling pathways.

## 5. Materials and Methods

All chemicals and culture media were purchased from Thermo Fisher Scientific (Waltham, MA, USA) unless otherwise. Porcine intestinal epithelial cell line IPEC-J2 (ACC-701, Leibnitz Institute DSMZ, German Collection of Microorganisms and Cell Cultures, Germany) were cultured in 1:1 DMEM (Dulbecco’s modified Eagle medium)/Ham’s F-12 mixture with 5% fetal bovine serum (FBS), antibiotic-antimycotic, 1% insulin-transferrin-selenium-X (ITS-X), and 5 ng/mL of epidermal growth factor at 37 °C in an atmosphere of 5% CO2. DON (D0156, Sigma-Aldrich, St. Louis, MO, USA) and LPS (L2630, Sigma-Aldrich, St. Louis, MO, USA) were dissolved in 0.1% acetonitrile and distilled water, respectively. For protein analysis, 4 × 10^5^ IPEC-J2 cells were seeded into each well of 6-well culture plates for 24 h and then treated with DON (1 µg/mL), LPS (100 ng/mL), or both at 37 °C at the indicated times (0.5, 1, and 2 h). Different concentration (0.1–0.3%) of nanoscale silicate platelets (NSP, J&A Technology, Taipei, Taiwan) and mixture of montmorillonites and yeast cell walls (MY, Life Rainbow Biotech Co., Ltd., Yilan, Taiwan) were pre-incubated with 1 µg/mL DON at 26 °C for 2 h in the water bath and the mixtures (DON and mycotoxin adsorbents) were then treated with IPEC-J2 cells (4 × 10^5^ cells/6-well culture plate) at 37 °C for 1 h. For mRNA analysis, 4 × 10^5^ IPEC-J2 cells were treated with DON (1 µg/mL), LPS (100 ng/mL), or both at 37 °C at the indicated times (6, 12, and 24 h). 0.3% NSP or MY were pre-incubated with 1 µg/mL DON at 26 °C for 2 h in the water bath and the mixtures (DON and mycotoxin adsorbents) were then treated with IPEC-J2 cells (4 × 10^5^ cells/6-well culture plate) at 37 °C. For optimization of signal detection in the quantitative reverse transcriptase-polymerase chain reaction (qPCR), IPEC-J2 cells were treated with mixtures (DON and mycotoxin adsorbents) for 6 h and 12 h for the inflammation-associated gene and tight junction-associated gene analysis, respectively. Cells in the control group (CR or untreated) were treated with 0.1% acetonitrile.

Total protein from cells was purified by radioimmunoprecipitation assay buffer containing phosphatase inhibitor cocktails (Merck Millipore, Burlington, MA, USA) and separated by sodium dodecyl sulfate-polyacrylamide gel electrophoresis, and then transferred to a polyvinylidine fluoride membrane (Merck Millipore, Burlington, MA, USA). The MAPK signaling pathway (p38, ERK, and JNK) primary antibodies (phosphor and total protein) were purchased from Cell Signaling Technology (Beverly, MA, USA). β-actin primary antibody (Cell Signaling Technology, Beverly, MA, USA) was used as a loading control of total protein. The secondary antibody coupled to horseradish peroxidase (Cell Signaling Technology, Beverly, MA, USA) was used in the chemiluminescence procedure (Immobilon Western, Merck Millipore, Burlington, MA, USA). The proteins on the membrane were visualized using a charge-coupled device digital camera (UVP ChemStudio PLUS Touch, Analytik Jena, Upland, CA, USA). The ratio of the intensity of bands corresponding to target protein per loading control was analyzed by densitometer software.

Cellular RNA was extracted and reverse-transcribed by using REzol (Protech Technology Enterprise Co., Ltd., Taipei, Taiwan) and iScript Reverse Transcription kit (Bio-Rad, Hercules, CA, USA), respectively. The qPCR was performed using iQ SYBR Green Supermix kit (Bio-Rad, Hercules, CA, USA) and CFX96 Touch Deep Well Real-Time PCR System (Bio-Rad, Hercules, CA, USA). PCR was executed by 40 cycles of 95 °C for 10 s and 55 °C for 30 s. The sequence of primers for qPCR is listed in Table 1. β-actin was determined as the internal control gene. Threshold cycle (Ct) values were obtained and relative gene expression was calculated according to the 2^−∆∆Ct^ formula.

All data were analyzed by one-way ANOVA through the general linear model procedure of SAS (SAS Institute, Cary, NC, USA). Means were compared using Tukey honestly significant difference test at a significance level of *p* < 0.05.

## Figures and Tables

**Table 1:** Primer sequences for qPCR.

Gene	GenBank Accession Number	Sequence (5′–3′)
*inos*	NM_001143690	F ^1^: ACCACGGAACCTAATGATGG
		R: GAGTTGGAGAGGGAGGGAGAT
*cox-2*	NM_214321	F: ATGATCTACCCGCCTCACAC
		R: AAAAGCAGCTCTGGGTCAAA
*il-6*	NM_214399	F: GCTATGAACTCCCTCTCCACA
		R: GCTATGAACTCCCTCTCCACA
*claudin 1*	NM_001244539	F: GATTTACTCCTACGCTGGTGAC
		R: CACAAAGATGGCTATTAGTCCC
*claudin 3*	NM_001160075	F: GCCAAAGCCAAGATCCTCTAC
		R: AGCATCTGGGTGGACTGGT
*occludin*	NM_001163647	F: GTAGTCGGGTTCGTTTCC
		R: GACCTGATTGCCTAGAGTGT
* β-actin *	XM_021086047	F: GCCAGGTCATCACCATCGG
		R: GTAGAGGTCCTTGCGGATGTC

^1^ F, forward primer; R, reverse primer.

## Data Availability

The data presented in this study are available on request from the corresponding author.
